# Supplementary material for: Arctic Ocean sea ice cover during the penultimate glacial and the last interglacial
Source: Nat Commun. 2017 Aug 29;8:373. doi: 10.1038/s41467-017-00552-1 (PMC5575311; doi:10.1038/s41467-017-00552-1)
Supplement: Supplementary file 1 — Supplementary Information [file 41467_2017_552_MOESM1_ESM.pdf]

Description of Supplementary Files

File Name: Supplementary Information
Description: Supplementary Figures, Supplementary Tables and Supplementary References

File Name: Peer Review File

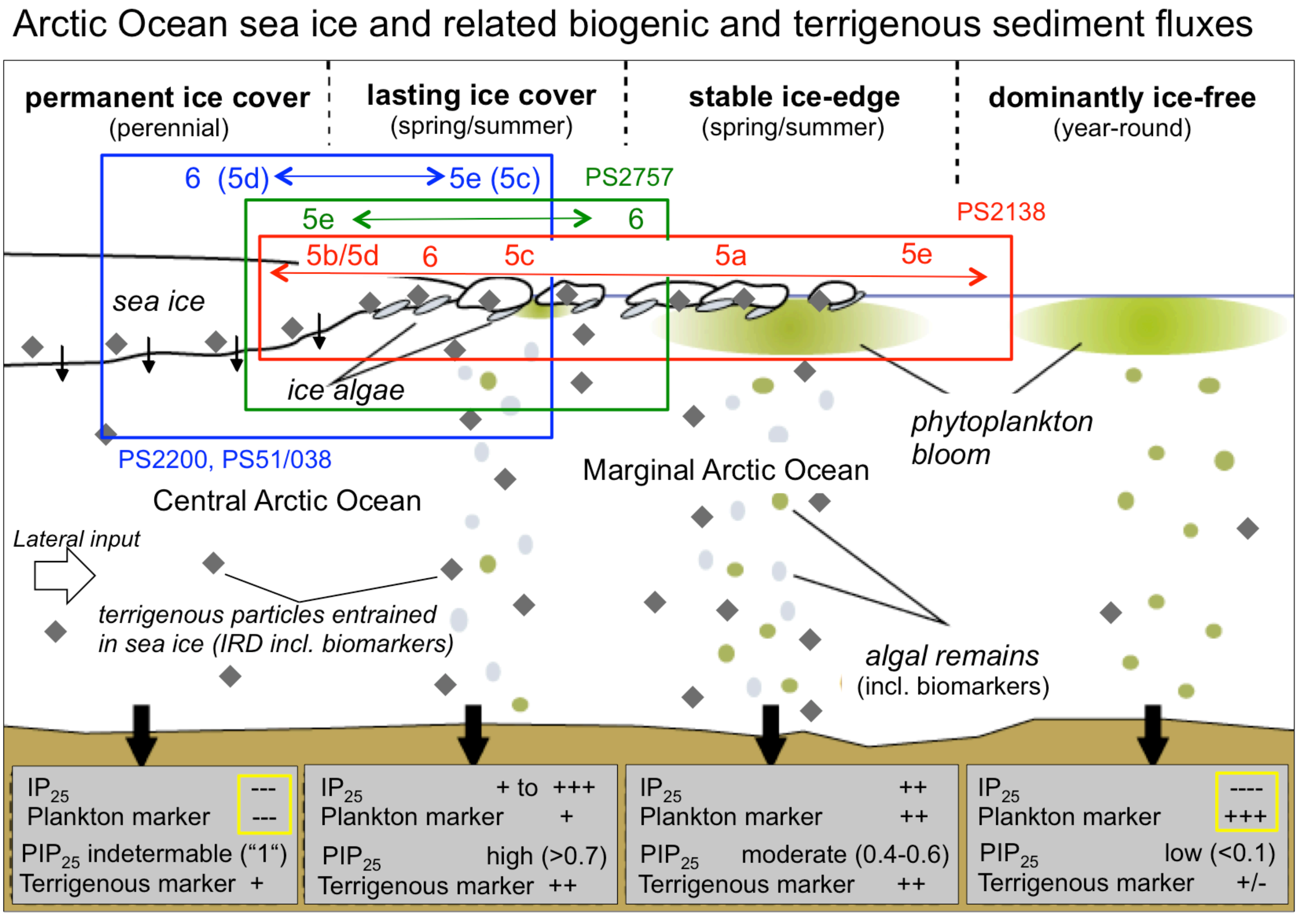

Supplementary Figure 1. Schematic illustration of Arctic sea-ice variability and associated biomarker records, and proposed MIS 6 – MIS 5 environmental conditions. Generalized scheme illustrating (1) sea surface conditions and respective (spring/summer) productivities of ice algae and phytoplankton, and (2) sedimentary contents of IP_{25}, phytoplankton-derived biomarkers, PIP_{25} index, and terrigenous biomarkers and ice-rafted debris (IRD) for different settings in the modern Arctic Ocean (after refs 1,2; supplemented). Due to ice melting and related nutrient and sediment release, a stable ice-edge situation is characterized by high concentrations of IP_{25}, phytoplanktion and terrigenous biomarkers as well as IRD. The proposed sea ice/ paleoenvironmental conditions during MIS 6 and MIS 5 (5a-5e) at the sites of the four studied sediment cores are indicated by blue (Cores PS2200-2 and PS51/38-3), green (Cores PS66/309-1 and PS2757-8) and red (Core PS2138-2) boxes.

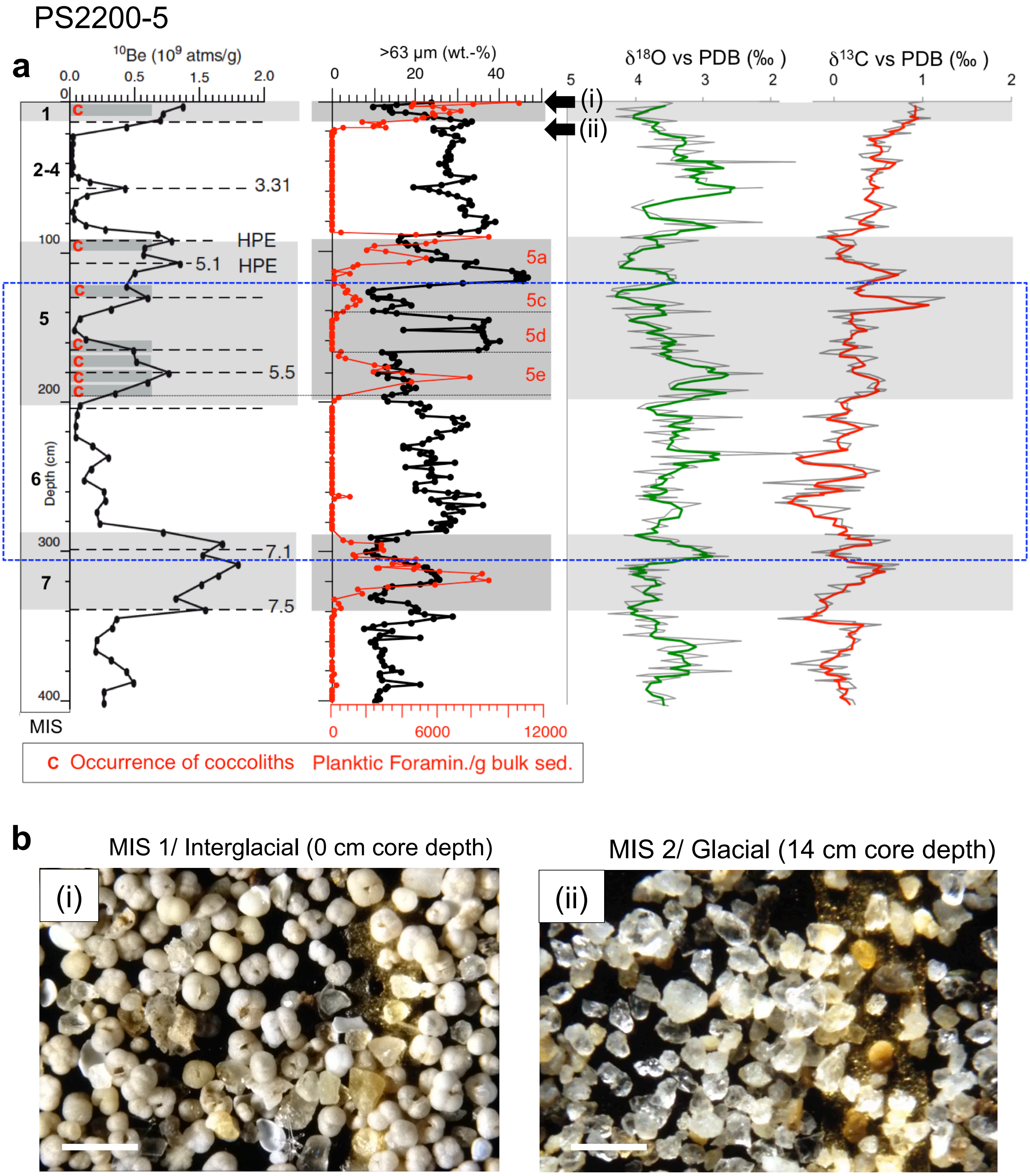

Supplementary Figure 2. Stratigraphic frame work and age model of Core PS2200-5. (a) ^{10}Be isotope, percentage >63 µm, number of planktic foraminifers per gram sediment records, and oxygen and carbon isotope records of planktic foraminifer *N. pachyderma* (sin.), representing the time interval from Marine Isotope Stage (MIS) 7 to MIS 1 (ref. 3). MIS events 7.5, 7.1, 5.5, 5.1 and 3.31 and high-production events (HPE) are indicated. Dark gray bars marked by "c" indicate occurrence of coccoliths. The box marked by the stippled blue line indicates the time interval represented by the biomarker record of Figure 2a. **(b)** Photographs of the coarse fraction >63 µm of a sample from 14 cm core depth (representing a glacial) and the surface sediment (representing an interglacial) are shown. White horizontal scale bar represents 500 µm.

Core PS51/038-3

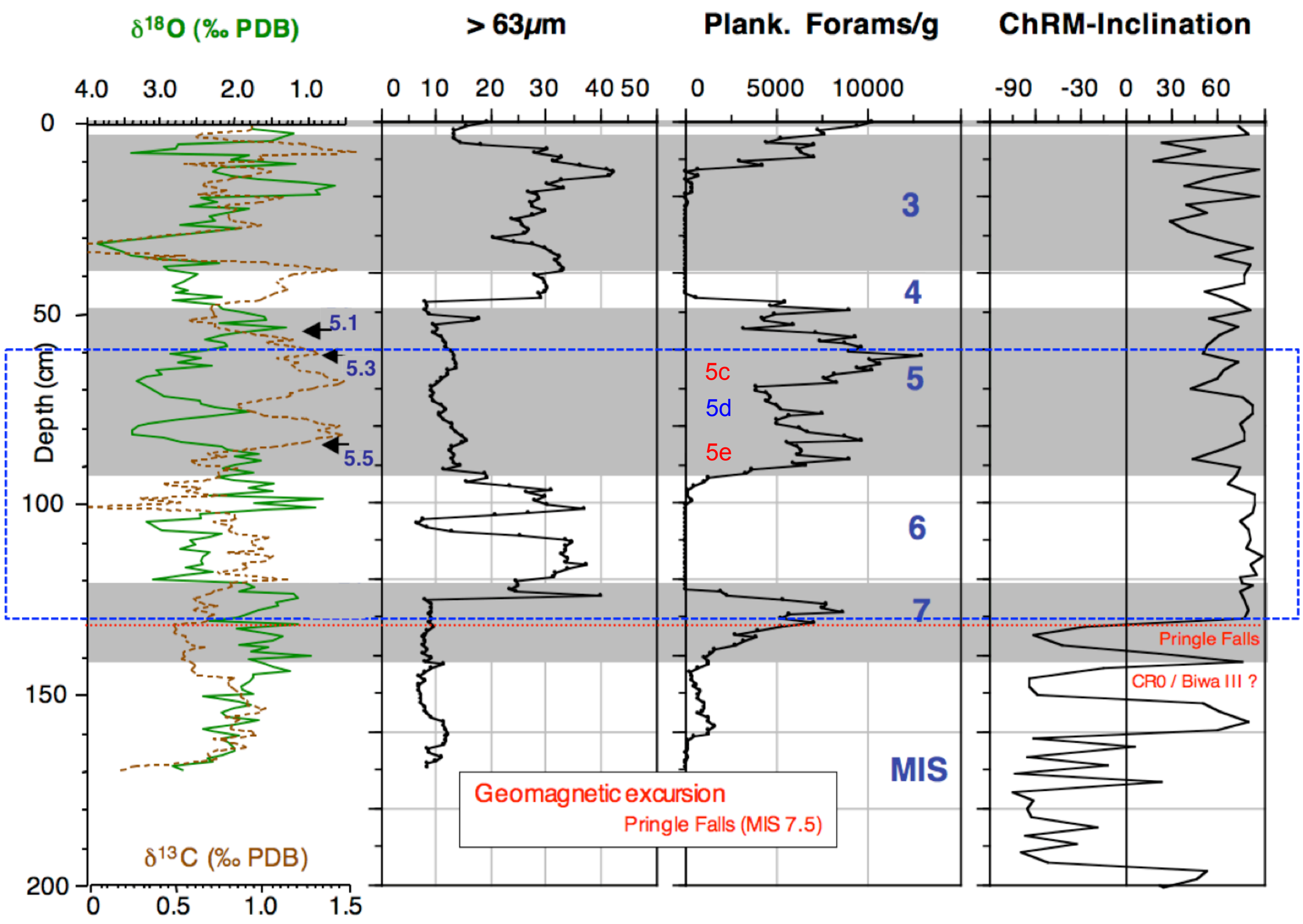

Supplementary Figure 3. Stratigraphic frame work and age model of Core PS51/038-3. Oxygen and carbon isotope records of planktic foraminifer *N. pachyderma* (sin.), percentage >63 µm, number of planktic foraminifers per gram sediment, and magneto-stratigraphy, representing the time interval from MIS 7 to MIS 1 and the geomagnetic events Pringle Falls and CR=/Biwa III (refs 3,4). IChRM = Inclination of the characteristic remanent magnetization. The box marked by the stippled blue line indicates the time interval represented by the biomarker record of Figure 2b.

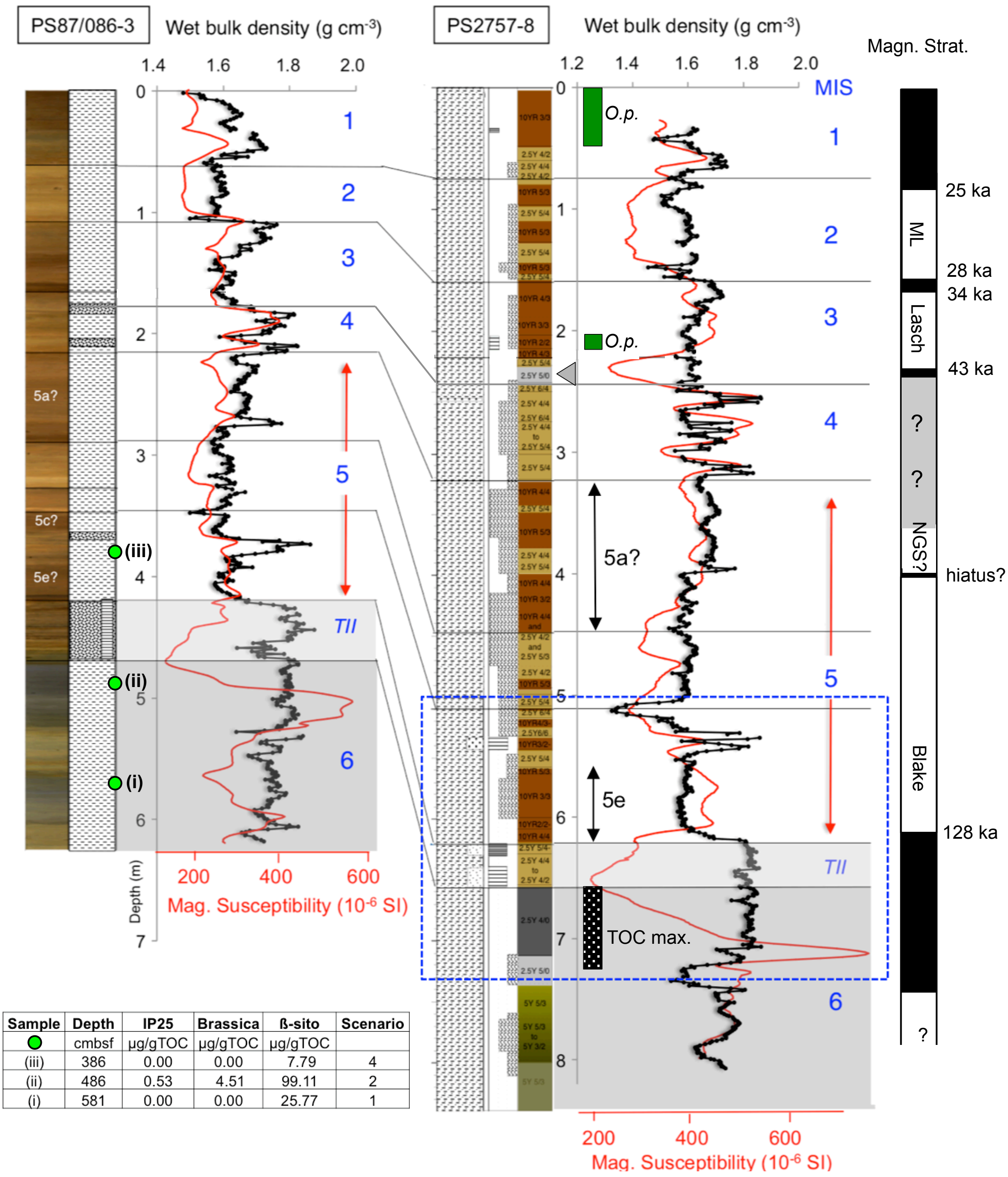

Sample	Depth	IP25	Brassica	ß-sito	Scenario
●	cmbsf	µg/gTOC	µg/gTOC	µg/gTOC	
(iii)	386	0.00	0.00	7.79	4
(ii)	486	0.53	4.51	99.11	2
(i)	581	0.00	0.00	25.77	1

Supplementary Figure 4. Stratigraphic frame work and age model of Core PS2757-8. Generalized columns of lithology and color, wet-bulk density and magnetic susceptibility records from Core PS57-8 and near-by Core PS87/086-3 and proposed isotope stratigraphy for the time interval from MIS 6 to MIS 1 (ref 5; supplemented). The age model of Core PS2757-8 is supported by dinocyst data – dinocyst peak abundance/occurrence of *Operculodinium centrocarpum* (green bar O.p.) - allowing the identification of MIS 3 and MIS 1 (ref. 6) and magnetostratigraphy shown as black & white (= normal & reversed polarity) column (N. Nowaczyk in ref. 7). Ages of geomagnetic reversals (ML = Mono Lake; Lasch = Laschamp; NGS = Norwegian Sea; and Blake Event)[8,9]. The upper MIS 6 sediments are characterized by maximum total organic carbon (TOC) values (dotted black bar) that can be correlated to other dated Arctic Ocean cores[10]. The MIS 4/3 boundary is further supported by the occurrence of a prominent TOC-enriched gray layer (marked by gray triangle in the PS2757-6 lithology column) that has been described in several sediment cores along the Eurasian continental margin[9,10]. The box marked by the stippled blue line indicates the time interval represented by the biomarker record of Figure 2c. In the lower left corner, some biomarker data from Core PS87/086-3 (i.e., IP_{25}, brassicasterol and ß-sitosterol)[2] are shown, representing the scenarios 1, 2 and 4 of Figure 6.

Core PS2138-2

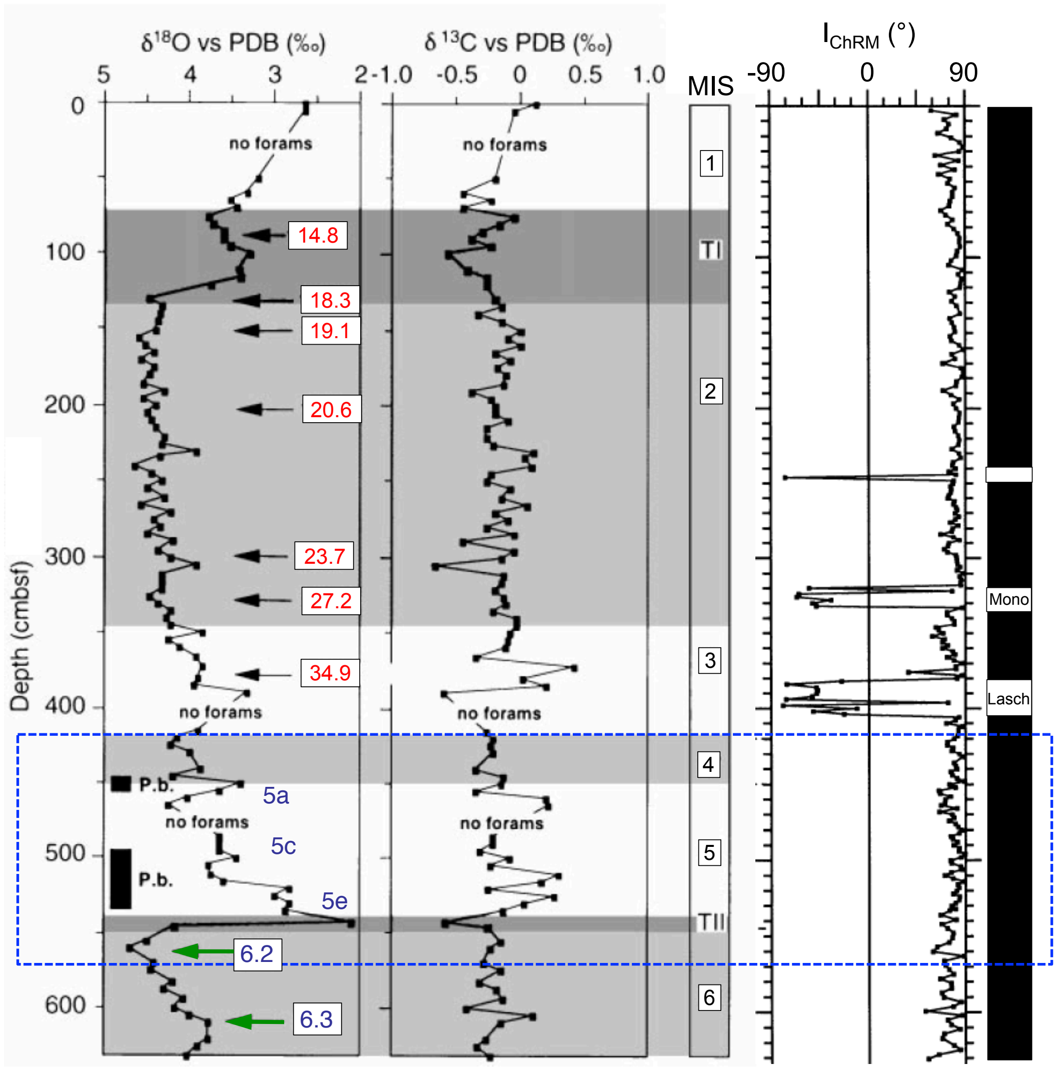

Supplementary Figure 5. Stratigraphic frame work and age model of Core PS2138-2. Oxygen and carbon isotope records of planktic foraminifer *N. pachyderma* (sin.), AMS^{14}C datings and magneto-stratigraphy, representing the time interval from Marine Isotope Stage (MIS) 6 to MIS 1 with terminations TII and TI and the geomagnetic events Laschamp and Mono Lake[9,11,12]. IChRM = Inclination of the characteristic remanent magnetization. The age model is mainly based on AMS^{14}C dating (black arrows with red numbers in Cal. kyrs. BP) for the upper about 400 cm of the record and the δ^{18}O record of *N. pachyderma* sin., supported by the occurrences of the benthic foraminifer *Pullenia bulloides* (black bars with “P.b. “) during substage MIS 5a and between substages MIS 5e and 5c (refs. 9, 13) and the identification of MIS events 6.2 and 6.3 (green arrows) according to ref 14. The box marked by the stippled blue line indicates the time interval, represented by the biomarker record of Figure 2d. The transformation of the depth scale of this interval into an age scale (see Fig. 7a-d) is based on ref 15.

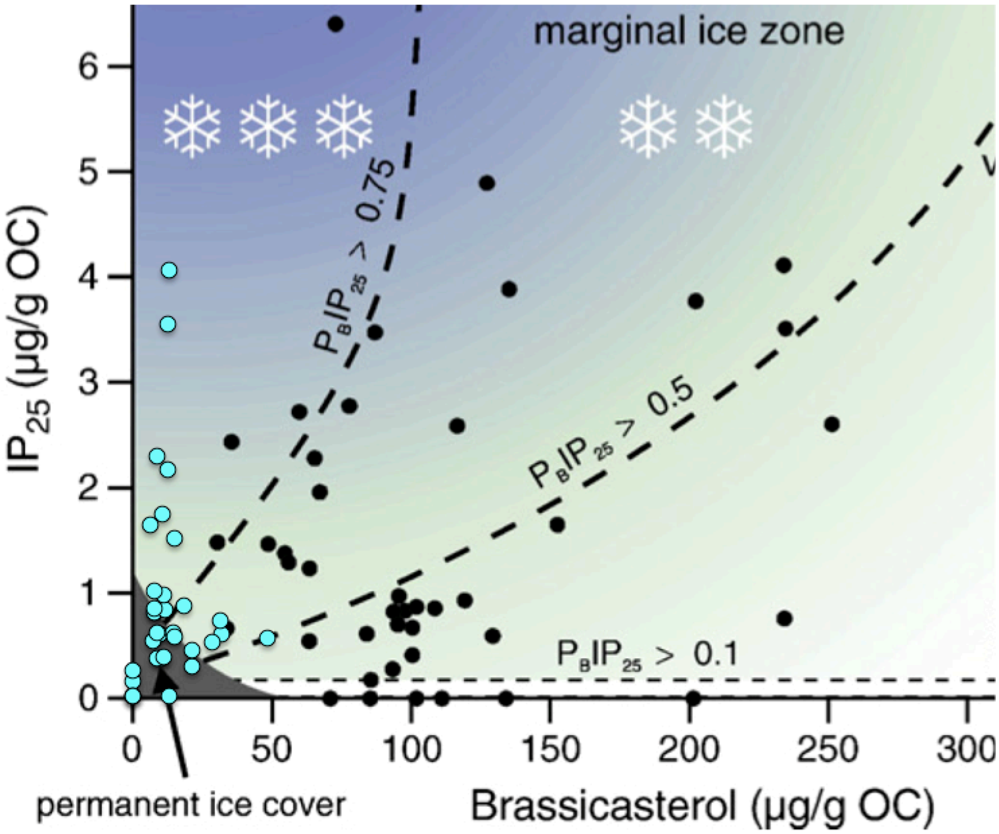

Supplementary Figure 6. Correlation of IP_{25} versus brassicasterol concentrations and corresponding P_BIP_{25} indices calculated for surface sediments (black circles) from the Fram Strait area (from the original work by Müller et al.[1]). The light-green to blue shaded background fields and snow symbols indicate the transition from minimum to maximum sea ice coverage. The dark grey field reflects samples from permanently sea ice-covered regions (perennial sea ice). Data obtained from surface sediments recovered from the central Arctic Ocean characterized by an extended to perennial sea ice cover, have been added (blue circles)[16]. Note that the absolute concentrations of IP_{25} and brassicasterol measured in these surface sediments are significantly higher than those determined in the MIS 1, LGM, MIS 5 and MIS 6 sediments (see Fig. 3). These differences are caused by early degradation of biomarkers in the uppermost centimetres of the sediments[17,18].

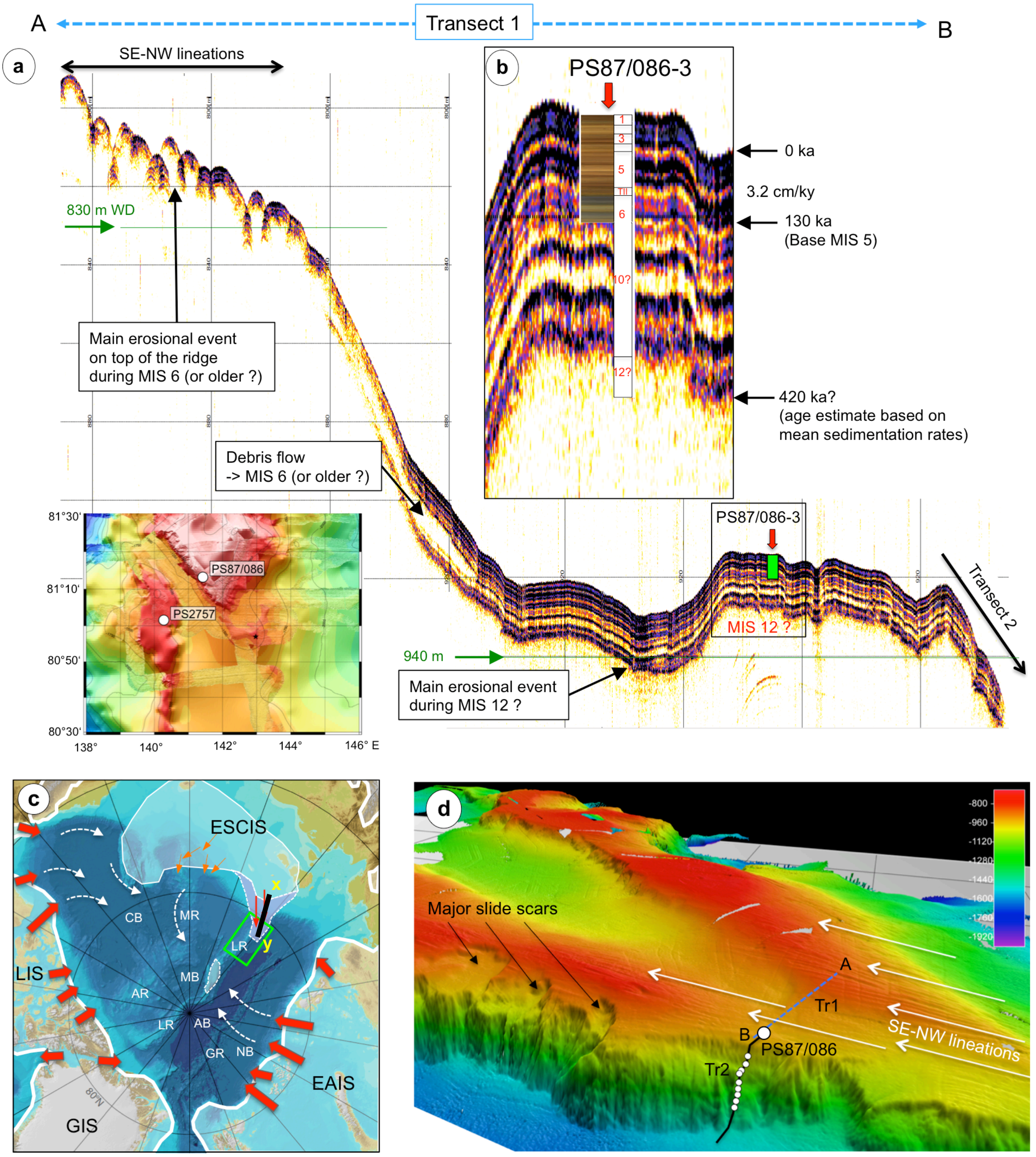

Supplementary Figure 7. (a) Parasound profile across southern Lomonosov Ridge with SE-NW oriented glacial lineations on the ridge crest (Transect Tr1, for location see (**c**)). The enlargement (**b**) shows area around Core PS87/086 as well as the core lithostratigraphy and proposed Marine Isotope Stages[5]. A debris flow on the mid slope is suggested to be related to glacial erosion on and sediment removal from the top of Lomonosov Ridge. Based on the correlation with the PS87/086 lithostratigraphy, the glacial erosion probably occurred during the MIS 6 glaciation. The transparent lower unit is separated from the stratified upper unit by an unconformity that may represent a major erosional event during a MIS 12 'mega glaciation' (age estimate based on extrapolation of the PS87/086-3 record). **(c) Proposed maximum extent of Quaternary circum-Arctic ice sheets**. Bathymetric map of the Arctic Ocean (IBCAO)[19] with distribution of Quaternary ice sheets; LIS – Laurentide Ice Sheet; GIS – Greenland Ice Sheet; EAIS – Eurasian Ice Sheet; ESCIS – East Siberian-Chukchi Ice Sheet[20-23]. Swath-bathymetry data from the study area indicates that the ESCIS extended further north on Lomonosov Ridge further to the north than proposed by Niessen et al.[23]. GR – Gakkel Ridge, LR – Lomonosov Ridge, AR – Alpha Ridge, MR – Mendeleev Ridge, CB – Canada Basin, MB – Makorov Basin, AB – Amundsen Basin, NB – Nansen Basin. Red arrows indicate ice-flow directions, white arrows indicate propagation of proposed ice shelves[22-24]. Black bar (x-y) indicates S-N transect shown in Fig. 6. **(d) 3D image of the swath bathymetry of southern Lomonosov Ridge**, showing major slide scars and escarpments, streamlined SE-NW oriented glacial lineations formed beneath grounded ice sheets/streams, and Transects Tr1 and Tr2 with locations of sediment cores[2].

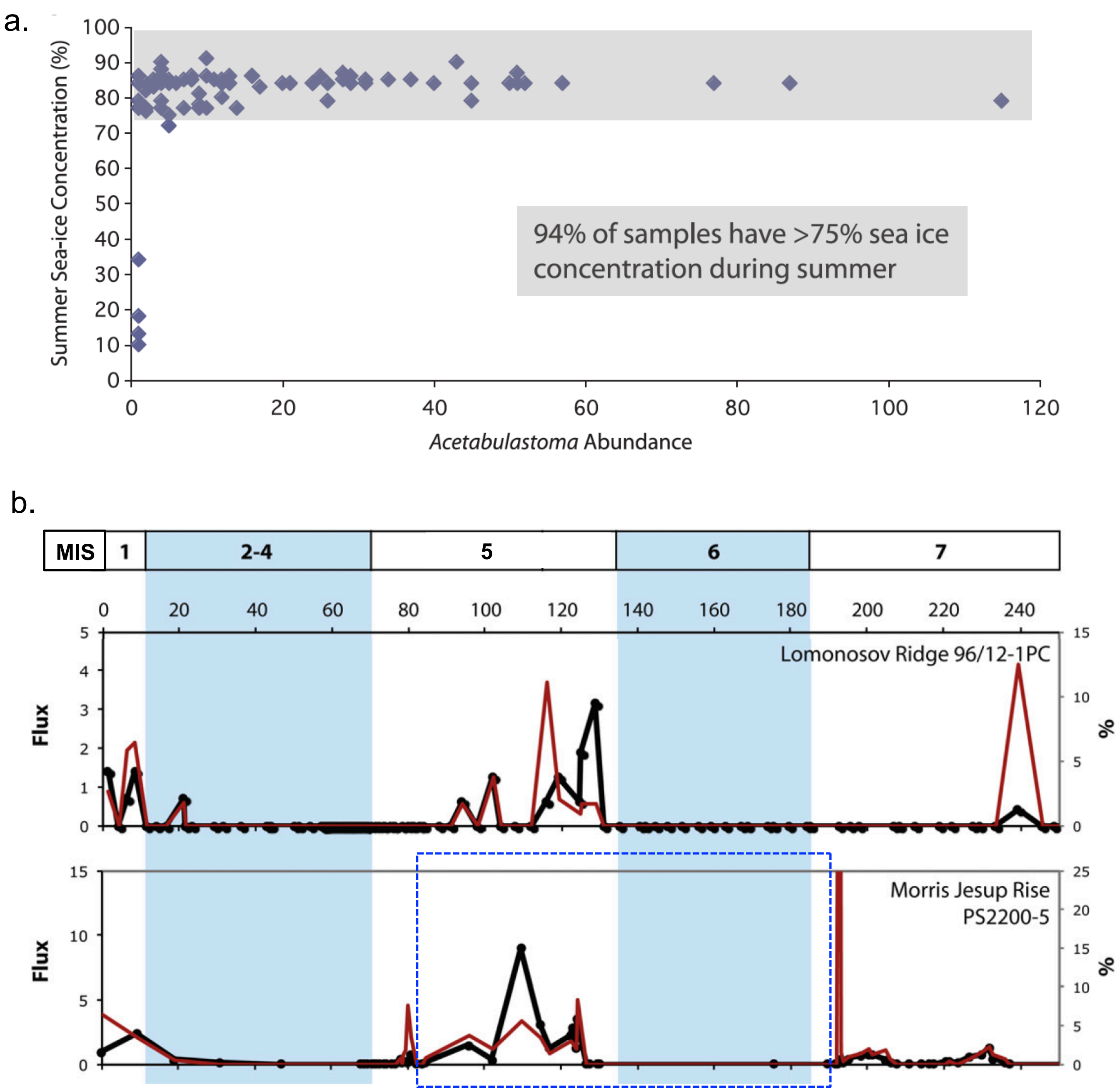

Supplementary Figure 8. Occurrence of sea-ice related ostracode species *Acetabulastoma arcticum* in surface sediments and two selected cores from the central Arctic Ocean[25].

a. Number of specimens of *A. arcticum* (abundance) plotted against mean September sea ice concentration from satellite records for the period 1978-1991 (http://nsidc.org/data/seaice/). 94% of samples yielding *A. arcticum,* are from central Arctic Ocean regions with perennial sea ice cover (i.e., >75% sea ice concentration). Unlike most ostracode species, *A. arcticum* is not benthic but rather lives parasitically on pelagic, sympagic (sea-ice associated) amphipods that inhabit Arctic Ocean areas with a perennial (permanent) sea ice cover today. Thus, *A. arcticum* seems to be a reliable proxy for perennial Arctic sea ice cover[25].

b. MIS 7 to MIS 1 records of flux of *A. arcticum* (individuals/gram/ky; black lines) and relative frequencies (% of total ostracodes; red lines) from two core sites from Lomonosov Ridge (Core 96/12-1PC; see Fig. 1 for location) and Morris Jesup Rise (PS2200-5)[25]. The box marked by the stippled blue line indicates the time interval represented by the biomarker record of Figure 2a.

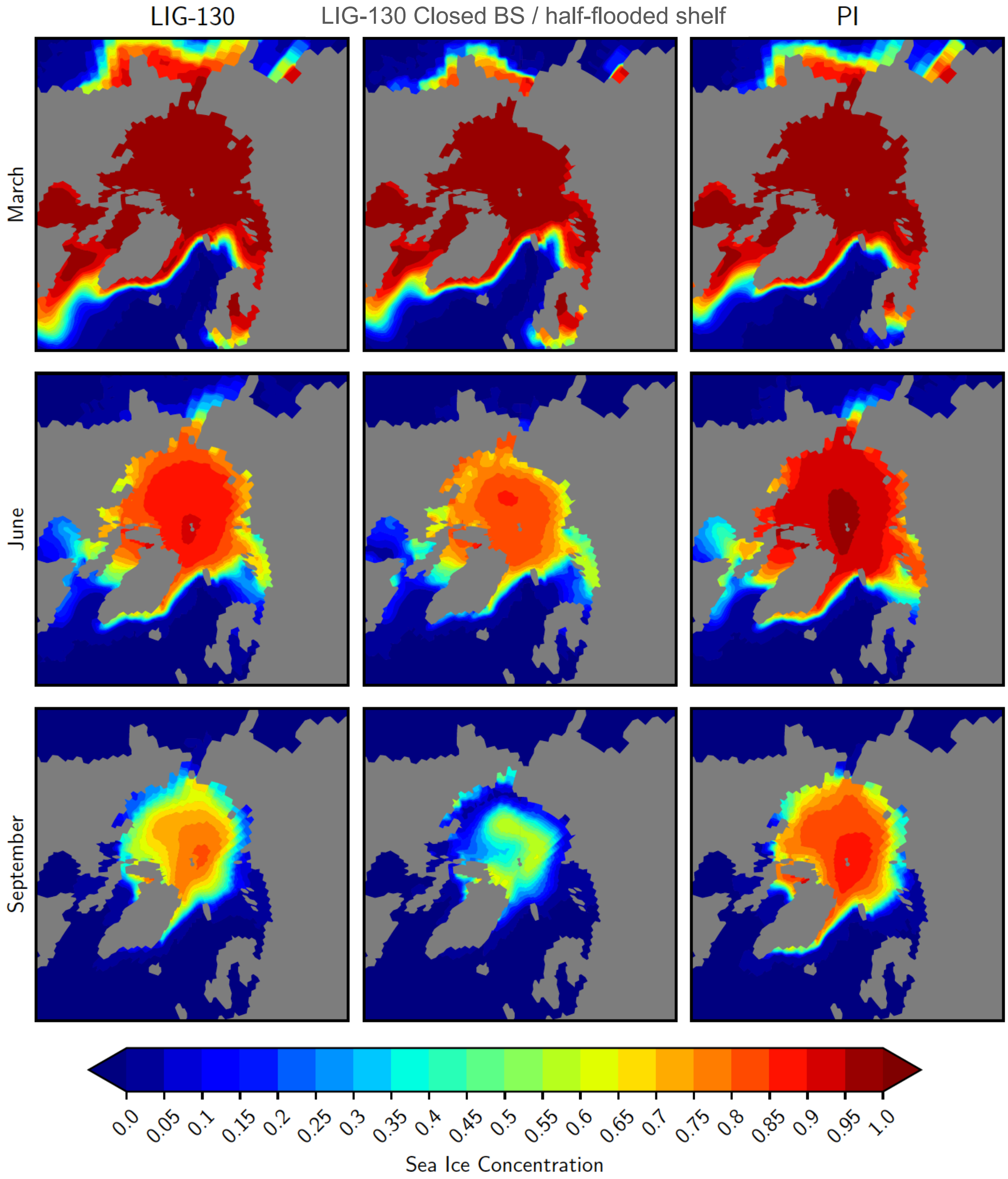

Supplementary Figure 9. Simulation of Arctic sea ice cover of two 130 ka (LIG-130) scenarios and the pre-industrial (PI) climate. LIG-130 conditions were simulated for (i) a scenario with an open Bering Strait and flooded shelf seas and (ii) a scenario with a closed Bering Strait (BS) and half-flooded Siberian shelf seas.

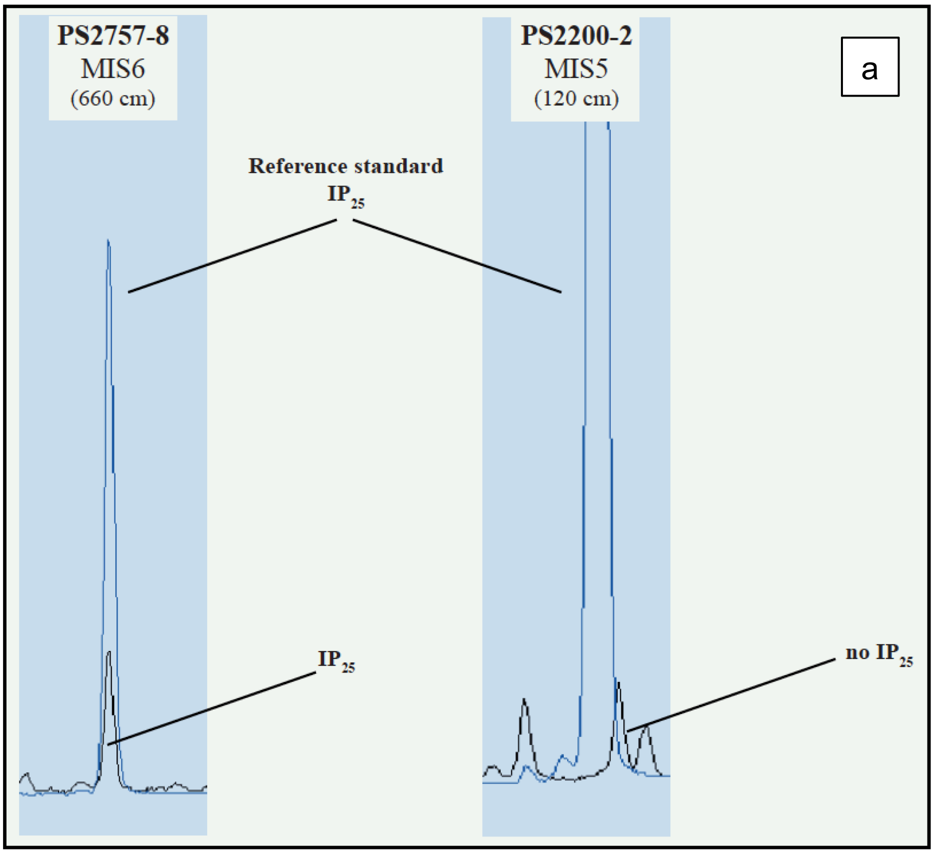

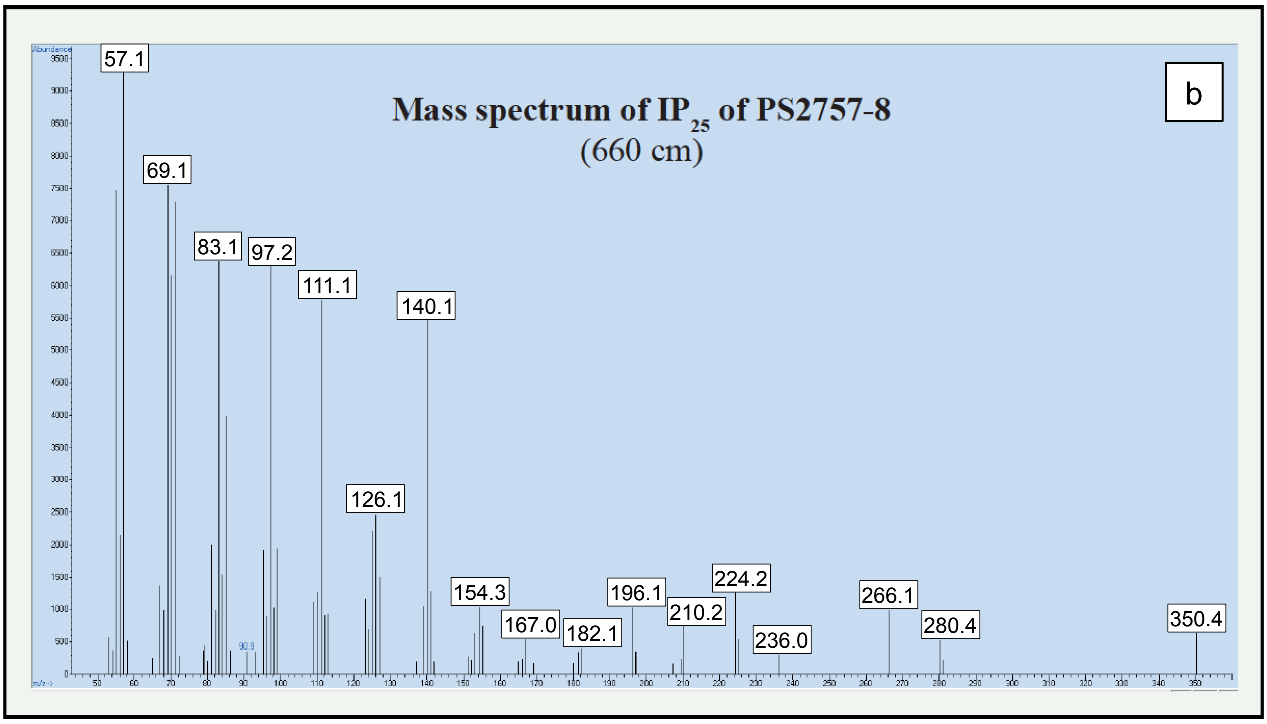

Supplementary Figure10. Identification of IP_{25}. (**a**) Selected ion monitoring chromatogram (SIM mode) of ion m/z 350 of the external IP_{25} standard and IP_{25} analysed in a MIS 6 sample from Core PS2757-8 (clear IP_{25} peak) and a MIS 5 sample from Core PS2200-2 (no IP_{25}). (**b**) Mass spectrum of IP_{25} in sample PS2757-8/660 cm.

Supplementary Table 1. Location and water depth of the four studied sediment cores as well as other cores mentioned in the text and/or shown on figures.

Core	Latitude	Longitude	Water depth (m)
PS2138-2	81.54 °N	30.59 °E	995
PS2757-8	81.19 °N	140.04 °E	1230
PS51/38-3	85.14 °N	171.43 °W	1473
PS2200-5	85.32 °N	14.00 °W	1073
PS2163	86.24 °N	59.22 °E	3075
PS2170	87.59 °N	60.77 °E	4112
PS2185	87.53 °N	144.17 °E	1052
PS2206	84.26 °N	02.56 °W	3020
PS2446	82.40 °N	40.90 °E	2022
PS2741	81.11 °N	105.39 °E	2530
PS2837	81.13 °N	02.38 °E	1042
PS87/086	81.22 °N	141.38 °E	901
MSM5/5-712	78.92 °N	06.77 °E	1487
NP05-11-70GC	78.40 °N	32.42 °E	293
GreenICE 10	84.80 °N	74.28 °W	1040

Supplementary Table 2. Biomarker data of Core PS2200-5 (Time interval MIS 6 - MIS5)

Depth	TOC	Campesterol	Campesterol	ß-Sitosterol	ß-Sitosterol	Dinosterol	Dinosterol	Brassicasterol	Brassicasterol	HBI-III (z)	HBI-III(z)	IP25	IP25
(m)	(%)	(µg/gSed)	(µg/gTOC)	(µg/gSed)	(µg/gTOC)	(µg/gSed)	(µg/gTOC)	(µg/gSed)	(µg/gTOC)	(ng/gSed)	(µg/gTOC)	(ng/gSed)	(µg/gTOC)
120	0,15	0	0	0,0206	13,61	0	0	0	0	0	0	0	0
130	0,15	0	0	0,0136	8,97	0	0	0	0	0	0	0,073	0,0483
140	0,11	0	0	0,1095	98,89	0	0	0	0	0	0	0,032	0,0286
150	0,18	0	0	0,0164	9,02	0	0	0	0	0	0	0,019	0,0103
158	0,13	0	0	0,0132	10,43	0	0	0	0	0	0	0,015	0,0120
164	0,16	0	0	0,0170	10,87	0	0	0	0	0	0	0,010	0,0067
170	0,16	0	0	0,0314	20,15	0	0	0	0	0	0	0,101	0,0648
175	0,14	0	0	0,0143	9,90	0	0	0	0	0	0	0	0
180	0,13	0	0	0,0073	5,68	0	0	present	present	0	0	0,041	0,0319
184	0,18	0	0	0,0110	6,01	0	0	present	present	0	0	0,000	0,0000
187	0,15	0	0	0,0108	7,47	0	0	0	0	0	0	0,019	0,0128
190	0,11	0	0	0,0220	19,28	0	0	0	0	0	0	0,014	0,0122
196	0,14	0	0	0,3272	230,56	0	0	0	0	0	0	0	0
200	0,15	0	0	0,0159	10,48	0	0	0	0	0	0	0	0
205	0,15	0	0	0,0170	11,01	0	0	0	0	0	0	0	0
210	0,22	0	0	0,0135	5,99	0	0	0	0	0	0	0	0
214	0,17	0	0	0,0170	10,24	0	0	present	present	0	0	0	0
221	0,20	0	0	0,0188	9,22	0	0	0	0	0	0	0,005	0,0027
225	0,20	0	0	0,1264	64,44	0	0	present	present	0	0	0	0
230	0,18	0	0	0,0607	34,09	0	0	0	0	0	0	0,037	0,0207
235	0,14	0	0	0,0072	5,03	0	0	0	0	0	0	0	0
240	0,18	0	0	0,0163	9,18	0	0	0	0	0	0	0	0
245	0,19	0	0	0,0158	8,49	0	0	0	0	0	0	0	0
250	0,16	0	0	0,0225	14,22	0	0	0	0	0	0	0,166	0,1054
255	0,20	0	0	0,0900	44,61	0	0	0	0	0	0	0,005	0,0026
260	0,20	0	0	0,0599	30,00	0	0	0	0	0	0	0,046	0,0228
270	0,23	0	0	0,0638	27,75	0	0	0	0	0	0	0,059	0,0257
280	0,20	0	0	0,0804	39,58	0	0	0	0	0	0	0	0
289	0,16	0	0	0,0302	18,49	0	0	0	0	0	0	0	0
300	0,18	0	0	0,0146	8,23	0	0	0	0	0	0	0,019	0,0109

Note: concentrations in ng/g sediment

Supplementary Table 3. Biomarker data of Core PS51/38-3 (Time interval MIS 6 - MIS5)

Depth	TOC	Campesterol	Campesterol	ß-Sitosterol	ß-Sitosterol	Dinosterol	Dinosterol	Brassicasterol	Brassicasterol	HBI-III (z)	HBI-III(z)	IP25	IP25
(m)	(%)	(µg/gSed)	(µg/gTOC)	(µg/gSed)	(µg/gTOC)	(µg/gSed)	(µg/gTOC)	(µg/gSed)	(µg/gTOC)	(ng/gSed)	(µg/gTOC)	(ng/gSed)	(µg/gTOC)
60	0,13	0	0	0	0	0	0	0	0	0	0	0	0
63	0,13	0	0	0,0041	3,1474	0	0	0	0	0	0	0	0
66	0,12	0	0	0,0073	5,9944	0	0	0	0	0	0	0	0
71	0,30	0	0	0,0063	2,1021	0	0	0	0	0	0	0	0
75	0,12	0	0	0,0047	4,1044	0	0	0	0	0	0	0	0
79	0,11	0	0	0	0	0	0	0	0	0	0	0	0
82	0,12	0	0	0	0	0	0	0	0	0	0	0	0
83	0,13	0	0	0	0	0	0	0	0	0	0	0	0
85	0,12	0	0	0	0	0	0	0	0	0	0	0	0
86	0,13	0	0	0	0	0	0	0	0	0	0	0	0
87	0,13	0	0	0,0149	11,5415	0	0	0	0	0	0	0	0
88	0,12	0	0	0,0036	2,9233	0	0	0	0	0	0	0	0
91	0,13	0	0	0	0	0	0	0	0	0	0	0	0
94	0,13	0	0	0,0164	12,8360	0	0	present	present	0	0	0	0
95	0,15	0	0	0,0249	16,7548	0	0	present	present	0	0	0	0
96	0,14	0	0	0,0211	14,9677	0	0	present	present	0	0	0	0
98	0,14	0	0	0,0305	22,0806	0	0	0	0	0	0	0	0
100	0,15	0	0	0,0374	25,3492	0	0	0	0	0	0	0	0
102	0,16	0	0	0	0	0	0	0	0	0	0	0	0
104	0,23	0	0	0,0221	9,7972	0	0	0	0	0	0	0	0
106	0,13	0	0	0,0136	10,6231	0	0	0	0	0	0	0	0
108	0,12	0	0	0	0	0	0	0	0	0	0	0	0
110	0,14	0	0	0	0	0	0	0	0	0	0	0	0
112	0,13	0	0	0	0	0	0	0	0	0	0	0	0
114	0,13	0	0	0,0205	16,1433	0	0	0	0	0	0	0,0209	0,1646
116	0,12	0	0	0,0119	9,8358	0	0	0	0	0	0	0	0
118	0,14	0	0	0,0277	19,8562	0	0	0	0	0	0	0	0
120	0,12	0	0	0,0478	38,6766	0	0	0	0	0	0	0	0
122	0,12	0	0	0	0	0	0	0	0	0	0	0	0
123	0,12	0	0	0	0	0	0	0	0	0	0	0	0
126	0,12	0	0	0	0	0	0	0	0	0	0	0	0
130	0,19	0	0	0	0	0	0	0	0	0	0	0	0

Note: concentrations in ng/g sediment

Supplementary Table 4. Biomarker data of Core PS2757-8 (Time interval MIS 6 - MIS5)

Depth	TOC	Campesterol	Campesterol	ß-Sitosterol	ß-Sitosterol	Dinosterol	Dinosterol	Brassicasterol	Brassicasterol	HBI-III (z)	HBI-III(z)	IP25	IP25
(m)	(%)	(µg/gSed)	(µg/gTOC)	(µg/gSed)	(µg/gTOC)	(µg/gSed)	(µg/gTOC)	(µg/gSed)	(µg/gTOC)	(ng/gSed)	(µg/gTOC)	(ng/gSed)	(µg/gTOC)
506	0,22	0	0	0,0203	9,2100	present	present	0	0	0	0	0	0
515	0,21	0	0	0,0207	9,6575	0	0	0	0	0	0	0	0
525	0,19	0	0	0,0000	0,0000	0	0	0	0	0	0	0	0
532	0,15	0	0	0,0168	10,9719	0	0	present	present	0	0	0	0
535	0,28	0	0	0,0196	6,9606	0	0	present	present	0	0	0	0
540	0,19	0	0	0,0000	0,0000	0	0	0	0	0	0	0,447	0,2393
545	0,22	0	0	0,0253	11,3338	0	0	present	present	0	0	0	0
550	0,24	0	0	0,0059	2,5157	present	present	present	present	0	0	0	0
555	0,23	0	0	0,0181	7,9829	0	0	present	present	0	0	0	0
560	0,18	0	0	0,0184	10,4436	0	0	present	present	0	0	0	0
565	0,20	0	0	0,0130	6,3420	0	0	present	present	0	0	0	0
570	0,21	0	0	0,0111	5,3118	0	0	present	present	0	0	0	0
575	0,21	0	0	0,0305	14,7070	0	0	present	present	0	0	0	0
580	0,30	0	0	0,0218	7,3858	0	0	present	present	0	0	0	0
585	0,24	0	0	0,0394	16,5736	0	0	present	present	0	0	0	0
590	0,23	0	0	0,0219	9,5538	0	0	present	present	0	0	0	0
595	0,23	0	0	0,0113	4,9349	0	0	present	present	0	0	0	0
600	0,25	0	0	0,0265	10,5371	0	0	0	0	0	0	0,040	0,0157
605	0,25	0	0	0,0273	10,9771	0	0	0	0	0	0	0,040	0,0160
610	0,28	0	0	0,0683	24,6242	0	0	present	present	0	0	0,519	0,1871
615	0,23	0	0	0,0406	17,9270	0	0	present	present	0	0	1,258	0,5561
620	0,18	0	0	0,0347	18,8872	0	0	present	present	0	0	0,014	0,0076
625	0,19	0	0	0,0302	15,5227	present	present	0,0027	1,3879	0	0	0	0
630	0,24	0	0	0,0147	6,0753	present	present	0,0025	1,0169	0	0	0	0
635	0,20	0	0	0,1121	57,3823	present	present	0,0042	2,1514	0	0	0	0
640	0,25	0	0	0,2332	93,2599	present	present	0,0064	2,5465	0	0	0	0
645	0,37	0	0	0,1967	52,8098	present	present	0,0095	2,5518	0	0	0	0
650	0,50	0	0	0,5081	100,6293	0,0075	1,4924	0,0254	5,0285	0,153	0,0304	1,146	0,2270
655	0,54	0	0	0,5197	95,8676	0,0133	2,4498	0,0214	3,9507	0,224	0,0413	1,606	0,2963
660	0,57	0	0	1,0172	179,8152	0,0088	1,5565	0,0339	5,9951	0,255	0,0451	2,224	0,3931
670	0,56	0	0	0,3294	58,9324	0,0080	1,4281	0,0171	3,0544	0,279	0,0499	0,910	0,1629
680	0,66	0	0	1,0955	164,7693	0,0159	2,3892	0,0355	5,3394	0,573	0,0861	1,699	0,2556
690	0,66	0	0	1,4732	221,8464	0,0187	2,8196	0,0440	6,6282	0,255	0,0384	1,229	0,1850
700	0,67	0	0	1,4998	223,2103	0,0200	2,9747	0,0491	7,3147	0,066	0,0098	1,510	0,2248
710	0,28	0	0	0,0329	11,7124	0,0015	0,5185	present	present	0	0	0	0
720	0,30	0	0	0,0350	11,6390	0,0013	0,4259	0,0040	1,3339	0,297	0,0989	0	0
730	0,23	0	0	0,0467	20,1364	0,0017	0,7286	0,0033	1,4037	0,364	0,1569	0	0

Note: concentrations in ng/g sediment

Supplementary Table 5. Biomarker data of Core PS2138-2 (Time interval 60-140 ka)

Depth	Age	TOC	Campesterol	Campesterol	ß-Sitosterol	ß-Sitosterol	Dinosterol	Dinosterol	Brassicasterol	Brassicasterol	HBI-III (z)	HBI-III(z)	IP25	IP25	P(B)IP25	P(III)IP25	P(III)IP25
(m)	(ka)	(%)	(µg/gSed)	(µg/gTOC)	(µg/gSed)	(µg/gTOC)	(µg/gSed)	(µg/gTOC)	(µg/gSed)	(µg/gTOC)	(ng/gSed)	(µg/gTOC)	(ng/gSed)	(µg/gTOC)	c = 0.016	c = 1	c = 0.32
420	60,000	0,42	0,0070	1,6842	0,0130	3,1080	0,0017	0,3964	0,0193	4,6340	0	0	0	0	1	1	1
426	62,668	0,80	0,0258	3,2305	0,0787	9,8643	0,0271	3,4008	0,1225	15,3536	2,406	0,3015	3,447	0,4321	0,64	0,59	0,82
432	65,812	0,64	0,0239	3,7214	0,0637	9,9304	0,0219	3,4169	0,0828	12,9073	0,589	0,0917	1,019	0,1588	0,43	0,63	0,84
433	66,336	0,71	0,0305	4,3123	0,0797	11,2509	0,0274	3,8683	0,1340	18,9205	1,642	0,2319	2,374	0,3353	0,53	0,59	0,82
450	77,040	1,05	0,0275	2,6183	0,0678	6,4604	0,0369	3,5159	0,1066	10,1530	0	0	2,341	0,2230	0,58	1,00	1,00
455	80,659	0,54	0,0179	3,3237	0,0459	8,5332	0,0159	2,9529	0,0577	10,7311	0,640	0,1189	1,183	0,2201	0,56	0,65	0,85
460	84,181	1,05	0,0459	4,3724	0,0973	9,2625	0,0396	3,7757	0,1803	17,1757	25,095	2,3900	0,889	0,0847	0,24	0,03	0,10
464	86,963	0,32	0,0019	0,5960	0,0092	2,8723	0,0013	0,4148	0,0096	3,0105	0	0	0	0	1	1	1
470	91,080	0,51	0	0	0,0053	1,0455	0	0	0,0045	0,8762	0	0	0	0	1	1	1
479	97,256	0,54	0,0083	1,5508	0,0229	4,2500	0,0040	0,7421	0,0116	2,1532	0	0	0	0	1	1	1
480	97,943	0,83	0,0083	0,9966	0,0156	1,8801	0,0058	0,6983	0,0181	2,1790	0	0	0	0	1	1	1
485	101,374	0,90	0,0389	4,3246	0,1165	12,9435	0,0350	3,8908	0,1789	19,8708	14,114	1,5674	4,985	0,5536	0,64	0,26	0,52
490	103,471	0,67	0,0079	1,1741	0,0123	1,8295	0,0052	0,7760	0,0148	2,2030	0	0	0	0	1	1	1
496	105,589	0,61	0,0125	2,0440	0,0303	4,9458	0,0078	1,2700	0,0245	3,9944	0,584	0,0954	0	0	1	1	1
500	107,000	0,68	0,0059	0,8625	0,0153	2,2465	0,0065	0,9621	0,0288	4,2326	0	0	0,137	0,0201	1	1	1
505	108,175	0,85	0,0132	1,5575	0,0321	3,7744	0,0141	1,6535	0,0173	2,0364	0	0	0,131	0,0155	1	1	1
510	109,350	1,03	0,0161	1,5636	0,0424	4,1145	0,0178	1,7308	0,0571	5,5472	2,864	0,2781	0,906	0,0880	0,50	0,24	0,50
512	110,680	0,88	0,0303	3,4472	0,0824	9,3787	0,0279	3,1706	0,1054	11,9877	2,751	0,3130	2,260	0,2572	0,57	0,45	0,72
516	113,340	0,99	0,0453	4,5645	0,1137	11,4622	0,0393	3,9580	0,1934	19,5014	3,662	0,3692	1,497	0,1509	0,33	0,29	0,56
518	114,670	0,86	0,0412	4,7774	0,0942	10,9234	0,0335	3,8819	0,1954	22,6454	3,499	0,4055	1,061	0,1230	0,25	0,23	0,49
520	116,000	0,94	0,0585	6,2200	0,1039	11,0450	0,0349	3,7133	0,2630	27,9573	8,587	0,9129	1,166	0,1239	0,22	0,12	0,30
528	121,333	0,91	0,0677	7,4118	0,1302	14,2397	0,0324	3,5428	0,3163	34,5996	23,272	2,5461	1,489	0,1629	0,23	0,06	0,17
530	122,667	0,85	0,0531	6,2630	0,1046	12,3384	0,0376	4,4383	0,2590	30,5334	9,490	1,1188	0,836	0,0986	0,17	0,08	0,22
535	126,000	0,85	0,0493	5,7666	0,1513	17,7078	0,0261	3,0563	0,2586	30,2600	10,887	1,2738	1,607	0,1881	0,28	0,13	0,32
545	132,517		0	0	0,0120	3,4700	0	0	0	0	0	0	0	0	1	1	1
555	134,239	0,57	0,0514	9,0704	0,0908	16,0289	0,0263	4,6444	0,1119	19,7493	3,128	0,5520	0	0	0,00	0,00	0,00
560	135,100	0,38	0,0165	4,2927	0,0502	13,0783	0,0185	4,8279	0,0273	7,1214	0,771	0,2010	0	0	1	1	1
565	136,067	0,48	0,0215	4,4724	0,0736	15,2695	0,0282	5,8537	0,0721	14,9608	0,832	0,1727	3,660	0,7599	0,76	0,81	0,93
570	137,033	0,46	0,0477	10,2647	0,1230	26,4522	0,0435	9,3507	0,1249	26,8697	2,517	0,5413	1,510	0,3248	0,43	0,38	0,65
										mean 13.65		mean 0.71		mean 0.227			

Note: concentrations in ng/g sediment

Note: zero values not considered for calculation of mean values

Note: PIP25 set to "1" (see text forfurther explanation)

Supplementary Table 6: Orbital and Greenhouse Gas Forcings used for LIG Experiments

Experiment	CO2	CH4	NO2	Eccentricity	Obliquity	Precession
PI	280	760	270	0.016724	23.446	282.04
LIG-120	268	572	261	0.041090	22.998	209.04
LIG-125	276	640	263	0.040000	23.790	127.14
LIG-130	257	512	238	0.038209	24.246	48.320

Supplementary References

01. Müller, J. *et al.* Towards quantitative sea ice reconstructions in the northern North Atlantic: A combined biomarker and numerical modeling approach. *Earth Planet. Sci. Lett.* **306**, 137-148 (2011).
02. Stein, R. *et al.* Evidence for ice-free summers in the late Miocene central Arctic Ocean. *Nature Comm.***7,** 11148, doi: 10.1038/ncomms11148 (2016).
03. Spielhagen, R.F. *et al.* Arctic Ocean deep-sea record of Northern Eurasian ice sheet history. *Quat. Sci. Rev.* **23** (11-13), 1455-1483 (2004).
04. Stein, R. Arctic Ocean Sediments: Processes, Proxies, and Palaeoenvironment. *Developments in Marine Geology*, Vol. 2, Elsevier, Amsterdam, 587 pp. (2008).
05. Stein, R. (Ed.). The Expedition PS87 of the Research Vessel *Polarstern* to the Arctic Ocean in 2014, Reports on Polar and Marine Research 688, Bremerhaven, Alfred Wegener Institute for Polar and Marine Research 688, 273 pp (http://epic.awi.de/37728/1/BzPM_0688_2015.pdf) (2015).
06. Matthiessen, J., Knies, J., Nowaczyk, N.R. & Stein, R. Late Quaternary dinoflagellate cyst stratigraphy at the Eurasian continental margin, Arctic Ocean: indications for Atlantic water inflow in the past 150,000 years. *Glob. Planet. Change* **31**, 65-86 (2001).
07. Behrends, M. Reconstruction of sea-ice drift and terrigenous sediment supply in the Late Quaternary: Heavy-mineral associations in sediments of the Laptev-Sea continental margin and the central Arctic Ocean. *Reps. Pol. Res*. **310**, 167 pp (1999).
08. Nowaczyk, N.R., Frederichs, T.W., Eisenhauer, A. & Gard, G. Magnetostratigraphic data from Late Quaternary sediments from the Yermak Plateau, Arctic Ocean. Evidence for four geomagnetic polarity events within the last 170 ka of the Brunhes Chron. *Geophys. J. Int.* **117**, 453-471 (1994).
09. Knies, J., Müller, C., Nowaczyk, N., Vogt, C. & Stein, R. A multiproxy approach to reconstruct the environmental changes along the Eurasian continental margin over the last 150 kyr. *Mar. Geol.* **163**, 317-344 (2000).
10. Stein, R. *et al.* Accumulation of particulate organic carbon at the Eurasian continental margin during late Quaternary times: Controlling mechanisms and paleoenvironmental significance. *Glob. Plan. Change* **31**, 87-102 (2001).
11. Knies, J. & Stein, R. New aspects of organic carbon deposition and its paleoceanographic implications along the northern Barents Sea margin during the last 30,000 years. *Paleoceanography* **13**, 384-394 (1998).
12. Nowaczyk, N. & Knies, J. Magnetostratigraphic results from the eastern Arctic Ocean: AMS 14C ages and relative palaeointensity data of the Mono Lake and Laschamp geomagnetic reversal excursions. *Geophys. Journ. Int.* **140**, 185-197 (2000).
13. Haake, F.W. & Plaumann, U., 1989. Late Pleistocene foraminiferal stratigraphy on the Vøring Plateau, Norwegian Sea. *Boreas* **18**, 343–356 (1989).
14. Martinson, D.G. *et al.* Age dating and the orbital theory of the ice ages: Development of a high-resolution 0 to 300000 years chronostratigraphy. *Quat. Res*. **27**, 1–27 (1987).
15. Knies, J. & Vogt., C. Freshwater pulses in the eastern Arctic Ocean during Saalian and Early Weichselian ice-sheet collapse. *Quat. Res.* **60**, 243-251 (2003).
16. Xiao, X., Fahl, K., Müller, J. & Stein, R. Sea-ice distribution in the modern Arctic Ocean: biomarker records from Trans-Arctic Ocean surface sediments. *Geochim. Cosmochim. Acta* **155**, 16-29 (2015).
17. Fahl, K. & Stein, R. Modern seasonal variability and deglacial/Holocene change of central Arctic Ocean sea-ice cover: New insights from biomarker proxy records. *Earth Planet. Sci. Lett*. **351-352C**, 123-133 (2012).
18. Belt S.T. & Müller J. The Arctic sea ice biomarker IP_{25}: a review of current understanding, recommendations for future research and applications in palaeo sea ice reconstructions. *Quat. Sci. Rev.* **79**, 9–25 (2013).
19. Jakobsson, M. *et al.* The International Bathymetric Chart of the Arctic Ocean (IBCAO) Version 3. *Geophys. Res. Lett.* **39**, L12609, doi:10.1029/2012GL052219 (2012).
20. Ehlers, J. & Gibbard, P. L. The extent and chronology of Cenozoicglobal glaciation. *Quat. Internat.* **164-165**, 6-20 (2007).
21. Jakobsson, M. *et al.* An Arctic Ocean ice shelf during MIS 6 constrained by new geophysical and geological data. *Quat. Sci. Rev*. **29**, 3505-3517 (2010).
22. Jakobsson, M. *et al.* Arctic Ocean glacial history. *Quat. Sci. Rev*. **92**, 40–67 (2014).
23. Niessen, F. *et al.* Repeated Pleistocene glaciation of the East Siberian Continental Margin. *Nature Geoscience* **6**, 842-846 (2013).
24. Polyak, L., Edwards, M.H., Coakley, B.J. & Jakobsson, M. Ice shelves in the Pleistocene Arctic Ocean inferred from glacigenic deep-sea bedforms. *Nature* **410**, 453-459 (2001).
25. Cronin, T.M. *et al.* Quaternary Sea-ice history in the Arctic Ocean based on a new Ostracode sea-ice proxy. *Quatern. Sci. Rev.* **29**, 3415-3429 (2010).
